# Imbalance in the Gut Microbiota of Children With Autism Spectrum Disorders

**DOI:** 10.3389/fcimb.2021.572752

**Published:** 2021-11-01

**Authors:** Hongfang Ding, Xinhao Yi, Xiaohua Zhang, Hui Wang, Hui Liu, Wei-Wei Mou

**Affiliations:** ^1^ Department of Pediatrics, Shengli Oil Field Central Hospital, Dongying, China; ^2^ Department of Central Laboratory, Shengli Oil Field Central Hospital, Dongying, China

**Keywords:** gut microbiota, autism spectrum disorders, children, high−throughput sequencing, Firmicutes and Actinobacteria

## Abstract

**Background:**

Autism spectrum disorder (ASD) are complex behavioral changes manifesting early in childhood, which impacts how an individual perceives and socializes with others. The study aims to assess the disparities in gut microbiota (GM) amongst healthy controls and children with ASD.

**Methods:**

The study was performed on 25 children with ASD and 20 healthy children. Autistic symptoms were diagnosed and assessed with the Diagnostic and Statistical Manual for Mental Disorders and the Autism Treatment Evaluation Checklist (ATEC). Gastrointestinal (GI) symptoms were assessed with a GI Severity Index (GSI) questionnaire. The fecal bacteria composition was investigated by the high−throughput sequencing of the V3–V4 region of the 16S rRNA gene. The alpha diversity was estimated using the Shannon, Chao, and ACE indexes. The unweighted UniFrac analysis and the PCA plots were used to represent the beta diversity. LDA and LEfSe were used to assess the effect sizes of each abundant differential taxon.

**Results:**

Children with high GSI scores had much higher ATEC Total scores than those with lower GSI-scores. GI symptoms were strongly associated with symptoms of ASD. There was no difference in Chao, ACE, and Shannon indexes between ASD patients and healthy controls. Both groups showed a significant microbiota structure clustering in the plotted PCAs and significant differences in its composition at the family, order, genus, and phyla levels. There were also noteworthy overall relative differences in Actinobacteria and Firmicutes between both groups.

**Conclusions:**

This study shows the relationship between the clinical manifestations of Autistic symptoms and GI symptoms. ASD patients have dysbiosis of gut microbiota, which may be related to the onset of ASD. These findings may be beneficial for developing ASD symptoms by changing gut microbiota.

## Introduction

Autism Spectrum Disorders (ASD) refers to a neurodevelopmental set of lifelong complex disorders characterized by rigid or repetitive interests and behaviors and/or difficulties socializing (US APAD-TFAV, 2013). Epidemiological studies have found that the prevalence of ASD in children and adolescents has reached 0.2% to 2.7% in recent decades (Sun et al., 2013; Wan et al., 2013; Baio, 2014; Morales-Hidalgo et al., 2018). There are many theories on the pathogenesis of ASD, mainly focusing on genetic, epigenetic, and environmental factors (Hallmayer et al., 2011; Kim and Leventhal, 2015). Although there are still yet no approved drugs for the core symptoms of autism, effective treatments for ASD, however, include social therapy, speech, nutritional, and behavioral therapy (Lacivita et al., 2017). Therefore, there is an urgent need to strengthen the research on the prevention, pathogenesis, and treatment of ASD (Bauman, 2010; Buie et al., 2010).

Recent studies have found that patients with ASD suffer from recurrent gastrointestinal (GI) symptoms such as vomiting, constipation, bloating, diarrhea, and abdominal pain (Hsiao, 2014; Fulceri et al., 2016). Rigid, compulsive behavior, abnormal sleep or eating habits, and opposing behaviors are linked to GI disorders (Adams et al., 2011; Maenner et al., 2012; Peters et al., 2014). Studying the pathophysiology of GI symptoms in ASD may be relevant for early recognition of ASD and the treatment of ASD by improving GI symptoms.

The gut microbiota (GM) present in our GI tract affects the developmental function of the immune, metabolic, and nervous systems. Its number is not only ten times that of human cells, but also the number of genes contained is 150 times that of the human genome (Sandoval-Motta et al., 2017). Recently, GM has been considered as a possible way to affect the manifestation of symptoms of cognitive and neurodevelopmental disorders. Although the cause of these associated symptoms is unknown, these symptoms partly appear to be due to the changes in the GM. However, recent research on specific gut microbes using animal models has demonstrated therapeutic potentials for ASD (Buffington et al., 2016; Tabouy et al., 2018). The gut-brain interaction pathways (biochemical and cellular) provides a platform for healthy gut microbiota to influence the neurochemistry, function, gene expression, and the development of the brain. Therefore, recent studies suggest that gut microbiota can affect the biochemical and behavioral phenotype of the brain *via* microbiota-gut-brain (Jiang et al., 2017). In this study, we performed a study on children with ASD and healthy controls to determine the association of fecal microbiota with ASD.

## Method

### Study Participants

The Ethics Committee approved the research of the Shengli Oil Field Central Hospital. Patients included in the study all accepted to participate and also gave prior written informed consent to be included in the study. Patients were not on pre or probiotics, antibiotics, and/or any other routine medications for six months before enrollment.

ASD diagnoses were carried out according to the Diagnostic and Statistical Manual for Mental Disorders (Fifth Edition (DSM-5)), and the Autism Treatment Evaluation Checklist (ATEC) was used to quantify the severity of ASD (Rimland and Edelson, 2000; US APAD-TFAV, 2013). GI symptoms were assessed using a GI Severity Index (GSI) questionnaire (Schneider et al., 2006). Children in the control group were physically and mentally healthy with no attention deficit disorders, and other GI symptoms such as vomiting, constipation, bloating, abdominal pain, and diarrhea (**Table 1**).

**Table 1:** Characterization of participants in the study.

	ASD group	Controls
Total	25	20
Gender (n): Male/Female	21/4	12/8
Age (years)	5.7 ± 1.4	5.4 ± 1.8

### GI Symptoms

Parents and/or guardians filled out questionnaires on their wards GI status, according to a GI Severity Index. GI symptoms include irritability, flatulence, abdominal pain and tenderness, insomnia, stool smell and consistency, constipation, and diarrhea. Moderate to severe gastrointestinal dysfunction based on GSI score (The total score is between 0 and 17, with higher values corresponding to higher severity).

### Severity Index

Parents were asked to complete the DSM-5 and ATEC scores in order to diagnose and assess the symptom severity. ATEC is composed of four subscales, which are used to calculate a total score that ranges from zero to a 179: (1) Physical/Behavior/Health; (2) Cognitive/Awareness/Sensory; (3) Language/Speech/Communication; and (4) Sociability. A lower score indicates less severe symptoms, and a higher score correlates with more severe symptoms of ASD.

### Sample Extraction and Collection

Fresh fecal samples were collected from each participant for the fecal microbiota test. Briefly, parents collected a 5-10 g of the single fecal sample from each subject into a sterile 50mL conical tube with gloves and a sterile spoon. Fecal samples were then kept in each patient’s refrigerator (-20°C) until transported in a cold pack to the laboratory, and stored at -80°C before analysis.

### PCR Amplification and DNA Extraction

Microbial DNA was extracted from samples using the OMEGA-soil DNA Kit. The final DNA concentration and purification were determined by NanoDrop 2000 UV-vis spectrophotometer, and DNA quality was checked by 1% agarose gel electrophoresis. PCR reactions were conducted using the following program: 3 min of denaturation at 95°C, 27 cycles of 30 s at 95°C, 30s for annealing at 55°C, and 45s for elongation at 72°C, and a final extension at 72°C for 10 min. PCR reactions were performed in triplicate 20 μL mixture containing 4μL of 5 × FastPfu Buffer, 2 μL of 2.5 mM dNTPs, 0.8 μL of each primer (5 μM), 0.4 μL of FastPfu Polymerase and 10ng of template DNA. The PCR products were extracted from a 2% agarose gel, purified and quantified using QuantiFluor™-ST.

### Illumina MiSeq Sequencing

Purified amplicons were pooled on an Illumina MiSeq platform in a paired-end and an equimolar sequence.

### Sequence Data Processing

Raw fast files were quality-filtered and merged by FLASH with the following criteria: (i) Reads were truncated at any site receiving an average quality score <20 over a 50 bp sliding window; (ii) Primers were precisely matched; and (iii) Overlapped sequences >10bp were unified based on the particular overlapped sequence.

Operational taxonomic units (OTUs) were clustered with 97% similarity cutoff using UPARSE, and chimeric sequences were identified and removed using UCHIME. RDP Classifier analyzed the taxonomy of each 16S rRNA gene sequence. Alpha diversity was calculated by four different parameters (i) Observed species; (ii) Shannon Index; (iii) phylogenetic diversity. Distance matrices (Beta diversity) between samples were generated based on non-weighted (unweighted UniFrac) algorithms and reported according to principal coordinate analysis (PCA). The linear discriminant analysis effect size was used to differentiate the fecal microbial features.

### Statistical Analysis

The data were expressed as mean ± standard deviation (SD. Statistical analysis was performed with SPSS, while biochemical variables and anthropometric measurements were performed with Student’s t−test. The Wilcoxon rank-sum test was used to determine the bacterial compositional changes between both groups. Statistical significance was set at P < 0.05.

## Results

### GI Symptoms and Autism Severity

This study found that children with autism are accompanied by GI symptoms such as constipation, diarrhea, and abdominal pain, among which constipation is the most common. Children with higher ATEC scores have severe gastrointestinal symptoms. The GSI score of the ASD group is closely related to the total ATEC score (**Table 2**).

**Table 2:** ATEC scores and GSI scores for the ASD group.

	Low-GSI ASD group	High-GSI ASD group
GSI scores	3.7 ± 0.9	11.2 ± 2.1
ATEC scores	63.2 ± 12.4	83.7 ± 10.7
Speech/Language/Communication	9.4 ± 4.6	13.7 ± 6.9
Sociability	16.3 ± 6.1	21.4 ± 7.5
Sensory/Cognitive Awareness	13.3 ± 7.5	17.9 ± 6.3
Health/Physical Behavior	24.5 ± 10.7	34.7 ± 11.4

### The Phylogenetic (α-Diversity) Diversity Analysis (Within-Sample)

**Figure 1:**
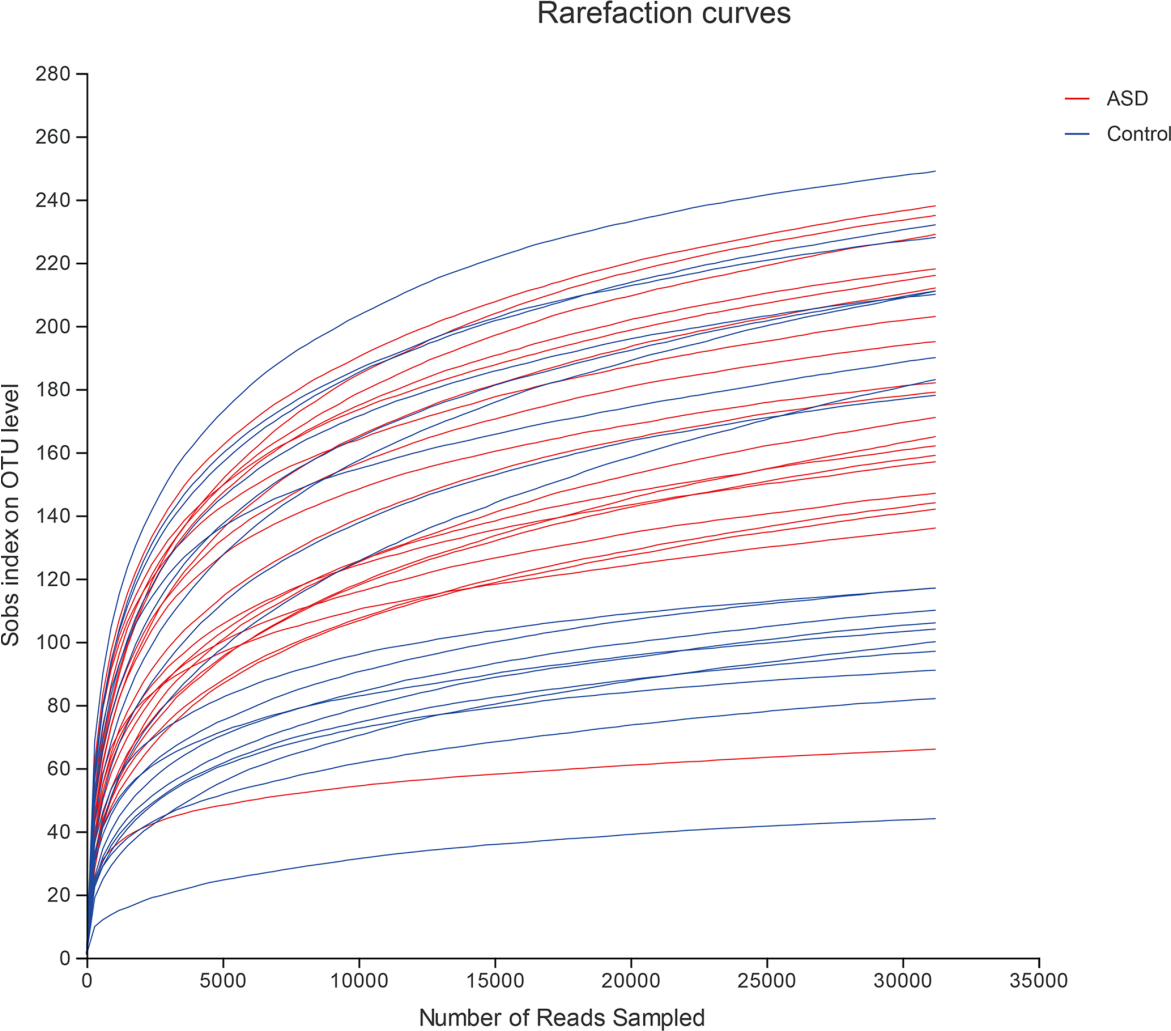
The bacterial community richness was analyzed by rarefaction curves (red: ASD group; blue: control group).

**Figure 2:**
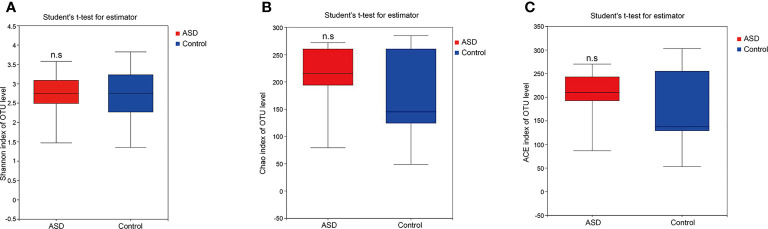
The within-sample phylogenetic diversity analysis (α-diversity): There was no significant differences of Chao1, ACE and Shannon indexes between healthy controls and ASD group (red: ASD group; blue: control group). **(A)** Chao index of OUT levels; **(B)** ACE index of OUT levels Sobs index of OUT levels; **(C)** Shannon index of OUT levels. n.s. represented “no significant”.

A sequence-based 16S ribosomal RNA gene method was used to correlate gut microbial communities in participants with ASD and healthy controls. High-quality sequences (useful reads ratio, ASD group 60.2%, Control group 62.4%) were produced in this study, which was afterward clustered into operational taxonomic units (OTUs) at a 97% similarity level. The vast majority of the OTUs, however, belonged to this phylogenic diversity (Actinobacteria, Proteobacteria, Bacteroidetes, and Firmicutes). **Figure 1** showed the bacterial community richness was analyzed by rarefaction curves. Diversity index (Shannon) and the OTUs estimators of the gut microbial communities (ACE and Chao1) are shown in **Table 3** and **Figure 2**. The Shannon index of the healthy control group was higher as compared to the ASD group, suggesting a higher species diversity. Also, the species richness and the ACE and Chao1indexes were higher in the healthy control group as compared to the ASD group; however, there were no statistical differences between the two groups.

**Table 3:** Chao1 and Shannon indexes in Controls and ASD group.

Items	Controls	ASD group	t	p
Chao	183.35 ± 76.792	215.63 ± 46.559	0.248	0.1189
Shannon	2.6679 ± 0.64353	2.7623 ± 0.49975	0.7328	0.6107

### The Degree of Microbial Phylogenetic Similarity Analysis (β-Diversity)

**Figure 3:**
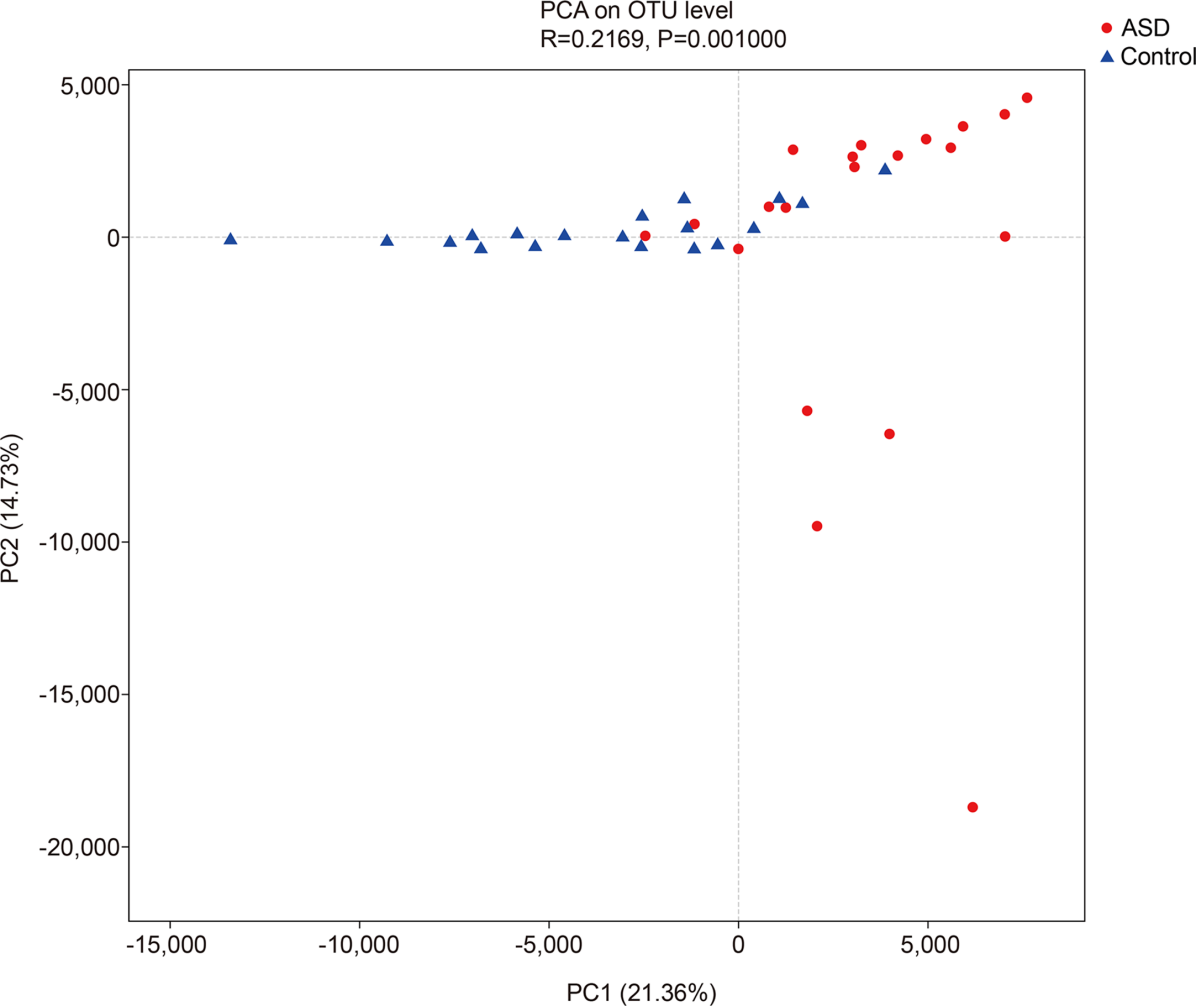
The unweighted UniFrac analysis which focuses on the degree of microbial phylogenetic similarity (β-diversity) in controls and ASD group (red: ASD group; blue: control group).

The unweighted UniFrac analysis which focuses on the degree of microbial phylogenetic similarity (β-diversity) – was used to determine the degree by which the gut microbiota within ASD subjects differed from those within healthy control. To compare the overall microbiota structure of the two groups, the unweighted UniFrac distance matrix was calculated based on the OTUs of each sample. The PCA plots based on distance revealed a significant separate clustering in microbiota structure between the two groups. The results were demonstrated by the first PC1 and the third PC2, accounting for 21.36% and 14.73% of total variations (**Figure 3**).

### Species Composition Analysis

**Figure 4:**
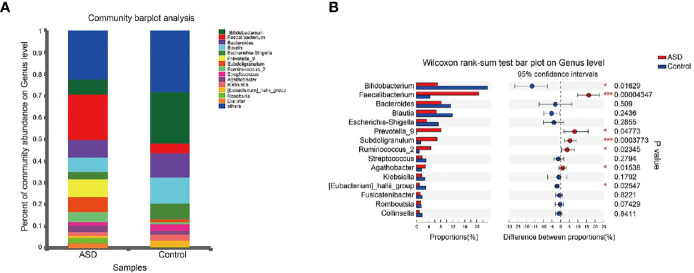
Microbial structures and the differences of genus between two groups (red: ASD group; blue: control group). **(A)** The community abundance of gut microbiota on genus level; **(B)** The difference of gut microbiota between ASD and control group on genus level. *P < 0.05 and ***P < 0.001.

A Random Forest classifier was applied, and a total of 637 OTUs, which could distinctively determine control samples from ASDs, were identified. A total of 555 OTUs and 541 OTUs, overrepresented in ASD participants, and healthy control patients were designated *Faecalibacterium*, *Bacteroides*, *Prevotella_9*, *Blautia*, *Subdoligranulum*, and *Bifidobacterium*, *Bacteroides*, and *Escherichia-Shigella* genera, respectively (**Figure 4**). Compared with healthy controls, *Faecalibacterium*, *Prevotella*, *Subdoligranulum* and *Ruminococcus* were more abundant in ASD patients, while *Bifidobacterium* was decreased. Other bacterial genus such as *Bacteroides*, *Blautia* and *Streptococcus*, exhibited no significant difference between ASD and controls.

**Figure 5:**
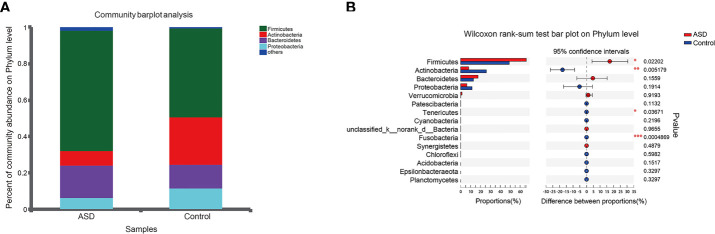
Relative abundance of fecalmicrobiota in each sample at the phylum level (red: ASD group; blue: control group). **(A)** The community abundance of gut microbiota on phylum level; **(B)** The difference of gut microbiota between ASD and control group on phylum level. *P < 0.05, **P < 0.01 and ***P < 0.001.

**Figure 6:**
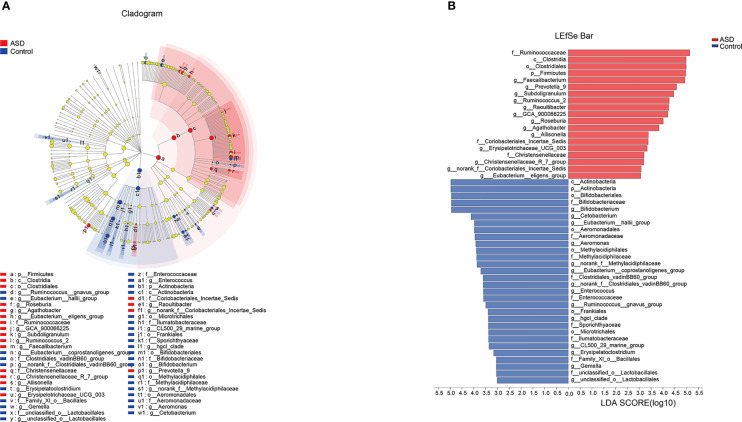
LEfSe was applied to assess the effect size of each differentially abundant taxon. LDA were performed to identify the most differentially abundant taxa. **(A)** Cladogram using results of the linear discriminant analysis (LDA) model on the bacterial hierarchy and **(B)** LDA coupled with effect size measurements identified the differentially abundant taxa between the two groups.

The discriminative OTUs were mainly, however, assigned to the phyla Actinobacteria, Firmicutes, and Bacteroidetes. The relative abundance of Firmicutes was lower in control than the test group, while Actinobacteria was decreased in ASD participants (**Figure 5**, P<0.05). Dominant phyla were Firmicutes, Actinobacteria, and Bacteroidetes, followed by Proteobacteria. To identify the specific bacteria taxa associated with ASD, we compared the GI microbiota in children with ASD with those in healthy controls using LEfSe. **Figure 6A** showed the cladogram that represents the phylogenetic structure of the GI microbiota and their predominant bacteria. The cladogram showed an apparent difference between the GI microbiota of the two groups. **Figure 6B** showed the LDA scores of these bacteria. Positive values (right) correspond to the effect sizes infants with ASD, and negative values (left) correspond to the effect sizes representative of controls. Ruminococcaceae and Firmicutes genus were found to be overrepresented in ASD cases, whereas Actinobacteria genus was overrepresented in healthy controls. Bacteria in different taxonomic levels that show a great difference and belong to the predominant phyla Firmicutes, Actinobacteria, and Bacteroidetes can be used as biomarkers to distinguish ASD.

## Discussion

Disruption of the microbiota-gut-brain axis through disturbances in GM has been suggested as a potential contributor to the emergence and development of ASD. The primary biological importance of the current study is associated with the lower level of healthy bacteria (e.g., *Bifidobacterium*) and the high *Faecalibacterium* and *Subdoligranulum* genera in ASD children. Some studies report that children with autism develop GI symptoms more frequently and more severely than children in the general population (Adams et al., 2011; Chaidez et al., 2014). In this study, it was found that children with ASD are accompanied by GI symptoms such as constipation, diarrhea, and abdominal pain, among which constipation is the most common, which is consistent with the results of other studies (Marler et al., 2017). This study also found a strong correlation between GSI scores for children with ASD and the Autism Treatment Evaluation Checklist score (Adams et al., 2011; McElhanon et al., 2014). Thus, it was concluded that the prevalence of GI symptoms was higher in children with ASD than in control children. In the treatment of children with ASD, the GI symptoms should be paid attention to and detected early. The presence of GI is an essential confounding factor in studying GM and ASD.

GM is considered a vital human organ and is affected by many factors during its formation. GM can also regulate intestinal mucosal epithelium development, provide resistance to potential pathogens, regulate gut lymphocyte function, and affect brain development and behavior (Heijtz et al., 2011). In recent years, emerging evidence suggests that altered gut microbiota plays a vital role in the development of various diseases, including schizophrenia, depression, and Parkinson’s disease (Dinan and Cryan, 2017). The intestinal microbiota plays a vital role in the health of an individual, such as trophic, metabolic, and protective functions. The gut microbiota may affect the central nervous system (CNS) through the gut-brain axis, which is a bidirectional link between the cognitive and emotional functions of the CNS and peripheral gut function (Grenham et al., 2011; Carabotti et al., 2015). The diverse mechanisms between the microbiome and the gut-brain axis are very complex (El Aidy et al., 2015; Koh et al., 2016; Sommer and Backhed, 2016). Gut microbiota and its metabolites and components affect host physiology by regulating intestinal barrier function, abnormal redox and mitochondrial metabolism, and mucosal inflammatory response (Hooper et al., 2012; Frye et al., 2013; Slattery et al., 2016).

In ecology, alpha diversity describes the richness and equitability of experiment sample species, and beta diversity is the extent of the differentiation of communities along habitat gradients. Our results showed that the species diversity of the GI microbiota of healthy controls in the alpha diversity category was higher than that of children with ASD, but no significant difference. Concerning beta diversity, the PCA plots based on distance revealed a notable separate clustering in the structure of the microbiota between the two groups.

Numerous studies have revealed the difference of the bacterial composition between ASD cases and controls. According to a systematic literature review at 2020 (Bundgaard-Nielsen et al., 2020), several studies reported increased relative abundance of *Bacteroides* (4 studies), *Barnesiella* (3 studies), *Clostridium* (4 studies), and *Roseburia* (3 studies), as well as reduced relative abundance of *Bifidobacterium* (5 studies), *Coprococcus* (3 studies), *Dialister* (3 studies), *Faecalibacterium* (4 studies), *Prevotella* (5 studies), and *Streptococcus* (4 studies) in cases. However, no specific bacteria consistently differed between ASD cases or controls in all of the included studies. Here in our studies, we concluded that *Faecalibacterium*, *Prevotella*, *Subdoligranulum* and *Ruminococcus* were more abundant in ASD patients, while *Bifidobacterium* was decreased. Other bacterial genus such as *Bacteroides*, *Blautia* and *Streptococcus*, exhibited no significant difference between ASD and controls. In summary, our results provided a new reference of bacterial composition of ASD cases.

Bifidobacteria, regarded as beneficial microbes, are typical gut inhabitants that belong to the phylum Actinobacteria (Ventura et al., 2007), which also improves intestinal function and immune modulation (Bottacini et al., 2014; Alessandri et al., 2019). These particular microbes have also been associated with the creation of several potentially health-promoting metabolites such as bacteriocins, short-chain fatty acids, and conjugated linoleic acid. Interestingly, studies have shown the beneficial complementary effects of bifidobacterial health-promoting advantages. Concerning brain-gut disorders, a recent experimental study has examined their psychobiotic effects in decreasing anxiety, stress, and other depressive behavior (Savignac et al., 2014). Nonetheless, in the study, the *Bifidobacterium* genus population was decreased in the ASD group as compared to the healthy control patient group. It may be inferred that children with ASD can be given a certain amount of Bifidobacterium to improve symptoms.

The reduced intestinal bacteria *Blautia* found in ASD patients is a butyric acid-producing bacterium that helps remove gas from the intestine and may be associated with irritable bowel syndrome (IBD) (McNabney and Henagan, 2017). In a previous study, *Blautia* was reported to have a significantly lower abundance of genus Blautia in ASD infants compared to healthy infants (Inoue et al., 2016), which is inconsistent with our results. In the study, *Subdoligranulum* was more prevalent in children with ASD. As fast as we know, no study have identified the difference of *Subdoligranulum* in ASD and controls. *Subdoligranulum* which contains the butyrate kinase gene is a direct source of energy source for colonic epithelium, and anti-inflammatory properties (vegetarian children and IBD) as a result of the inhibition of NF-κB (Chumpitazi et al., 2014; Matijasic et al., 2014; McNabney and Henagan, 2017). Compared to the control group, *Streptococcus* species were lower in ASD, which can produce lactate together with *Lactobacillus*, *Bifidobacterium*, and *Lactococcus* (Zhang et al., 2018).

This study showed that Actinobacteria, Firmicutes, and Bacteroidetes, which were the main bacterial phyla remarkably differed in the two groups. In the bacteria domain, Actinobacteria were the most populated and the most astronomical taxonomic units, followed by Proteobacteria and Firmicutes in early childhood. Firmicutes, Bacteroidetes, and Proteobacteria were the majority of the GI microbiota in humans in previous studies (Finegold et al., 2010; Kang et al., 2013). Faecalibacterium was present at a higher level in fecal samples of ASD children, and a trend can be seen that Firmicutes is much higher in the ASD group than controls. Bacteroidetes were lower in counts in ASD subjects than controls, but no statistical significance. Bacteroidales play a significant role in the fermentation of complex carbohydrates, which provide the host with nutrition and immune regulation (Mazmanian et al., 2005; Cuskin et al., 2015). Short-chain fatty acid and propionic acids were notably produced by a vast number of species in the Bacteroidetes as their metabolic end products, which provided the host with nutrition and immune regulation (Mazmanian et al., 2005; Cuskin et al., 2015). Some studies have shown that Bacteroidales are abundant in much gastrointestinal microbiota and immune deficiency or malnutrition diseases, such as colitis, hepatitis, and bowel cancer (Keren et al., 2015; Aly et al., 2016; Dubin et al., 2016). However, the other studies demonstrated that a low level of Bacteroidetes was associated with allergic disease (Abrahamsson et al., 2012).

Our results showed that there were lower abundances of the genera Bacteroidetes in health control and children with ASD. However, in other studies, Bacteroidetes often have a highest abundances in patients and health controls. The reason for the opposite results in different studies may be related to the age of participants, the geographical areas, sample location, and dietary habits (Qin et al., 2010; Claesson et al., 2011; Williams et al., 2011). These findings could be beneficial for establishing strategies to modify gut microbiota or other clinical methods to control the development of ASD. Some limitations of our study should be mentioned. First, relatively few samples carry a risk of failing to provide adequate information on the gut microbiota profile of ASD patiens. Second, 25 children with ASD and 20 healthy children originate from Chinese Han ethnicity, which is different from that of chip dataset samples (American population).

## Conclusion

In this study, we found a striking association between GI symptoms and ASD. We also found the diversity and composition of the intestinal flora in children with autism to be remarkably different from the control group. Therefore, we infer that the GM may be related to the etiology and pathophysiology of ASD, and can be used as a biomarker for ASD in the future. In different studies on the GM of ASD children, it has been found that the relevant intestinal microbial species are different. These different research results may have a common mechanism of action or pathway is also our future research focus.

## Data Availability Statement

The raw data supporting the conclusions of this article will be made available by the authors, without undue reservation.

## Ethics Statement

The studies involving human participants were reviewed and approved by Ethics Committee of the Shengli Oil Field Central Hospital. Written informed consent to participate in this study was provided by the participants’ legal guardian/next of kin.

## Author Contributions

Conception and design: XY and HD. Execution of experiments: HD, XZ and HW. Analysis and interpretation of data: HD, HW and HL. Writing, review, and/or revision of the manuscript: XY, W-WM and HW. All authors contributed to the article and approved the submitted version.

## Conflict of Interest

The authors declare that the research was conducted in the absence of any commercial or financial relationships that could be construed as a potential conflict of interest.

## Publisher’s Note

All claims expressed in this article are solely those of the authors and do not necessarily represent those of their affiliated organizations, or those of the publisher, the editors and the reviewers. Any product that may be evaluated in this article, or claim that may be made by its manufacturer, is not guaranteed or endorsed by the publisher.
